# Endoscopic intermuscular dissection of early anal cancer

**DOI:** 10.1055/a-2321-9527

**Published:** 2024-06-05

**Authors:** Hao Dang, Daan A. Verhoeven, Kirill Basiliya, Jurjen J Boonstra

**Affiliations:** 1 4501 Department of Gastroenterology and Hepatology, Leiden University Medical Center, Leiden, Netherlands

Anal cancer accounts for 0.5% of all new cancer cases, with an observed annual increase in incidence of up to 2.7% over the past decade 1 . This increase has closely mirrored the rise in human papillomavirus infections, the most important risk factor for anal cancer. According to international guidelines 2 3 4 , marginal/perianal lesions smaller than 2 cm without lymphatic involvement or metastatic spread can be curatively treated with complete local excision, thereby sparing patients the adverse effects of surgery or chemoradiotherapy. Here, we describe a case of early anal cancer which was successfully treated by endoscopic intermuscular dissection (EID).

A 44-year-old woman presented with rectal blood loss. Colonoscopy revealed a 30-mm laterally spreading polyp with a large nodule and involvement of the dentate line. Endoscopic assessment showed an unusual pit pattern on the top of the large nodule ( Fig. 1 ). Virtual chromoendoscopy showed nonstructured, amorphous pits and nearly avascular and loose microcapillary vessels ( Fig. 2 ). As deep submucosal invasion was suspected, EID was performed (see step-by-step explanation in Video 1 ). We used a conventional video endoscope (GIF-TH190; Olympus, Germany) with a small-caliber-tip transparent hood (DH-28GR; Fujifilm, Japan) fitted to the tip of the endoscope. A FlushKnife BT (DK2618JB-15; Fujifilm, Japan) was used for incision and dissection. For electrical cutting and coagulation, a VIO 300D electrosurgical generator (Erbe Elektromedizin, Germany) was used. EID was carried out using the tunneling method 5 : an intermuscular tunnel was created from the anal canal to the proximal side in the distal rectum, followed by mobilization of the lateral edges. Complete en bloc resection was achieved ( Fig. 3 , Fig. 4 ; total procedure time 120 min). Histological analysis showed a T1Sm2 squamous cell carcinoma with free resection margins (>2 mm) and no signs of lymphovascular invasion or high-grade tumor budding ( Fig. 5 ).

**Fig. 1:**
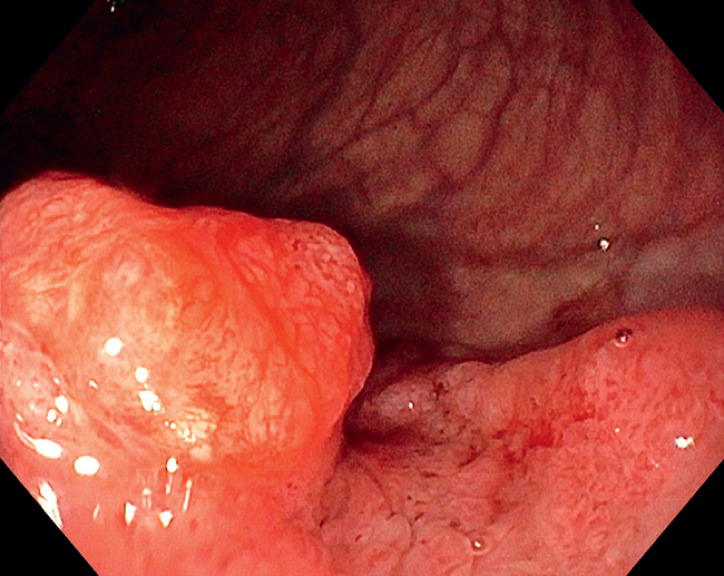
White-light imaging of the top of a large anal nodule showing an unusual pit pattern in a 44-year-old woman presenting with rectal blood loss.

**Fig. 2:**
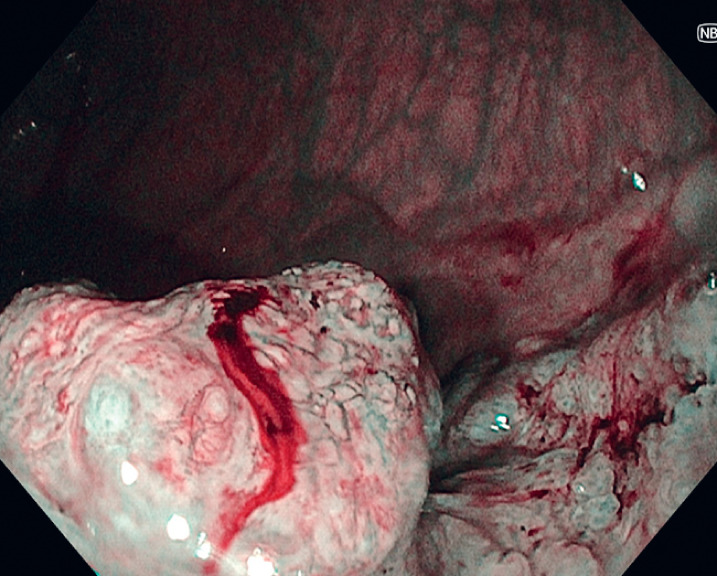
Narrow-band imaging of the top of the large nodule showing nonstructured, amorphous pits and nearly avascular and loose microcapillary vessels.

**Fig. 3:**
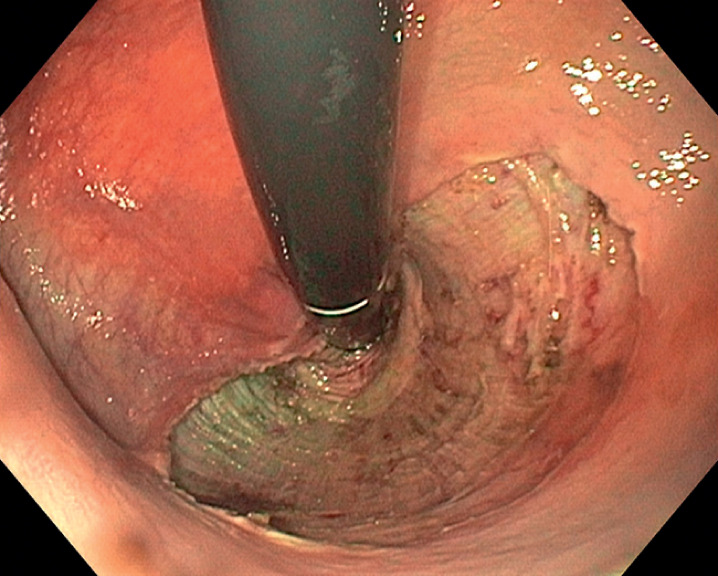
Endoscopic view of the resection site after endoscopic intermuscular dissection (EID).

**Fig. 4:**
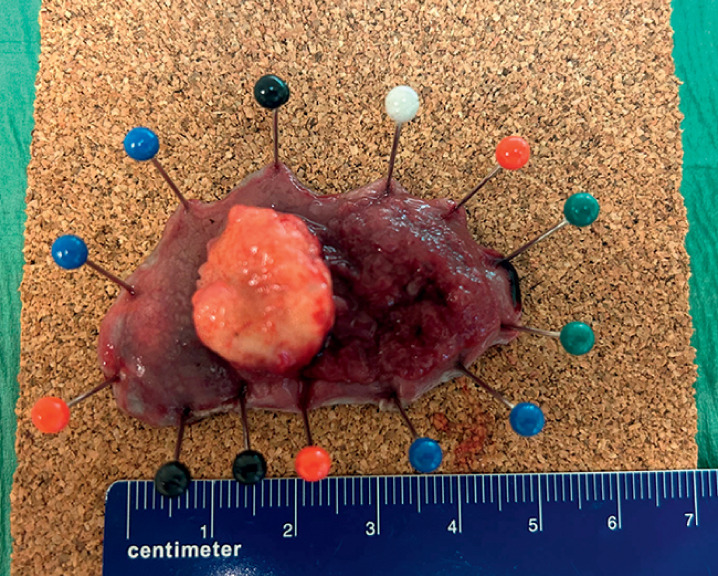
Macroscopic view of the resected specimen.

**Fig. 5:**
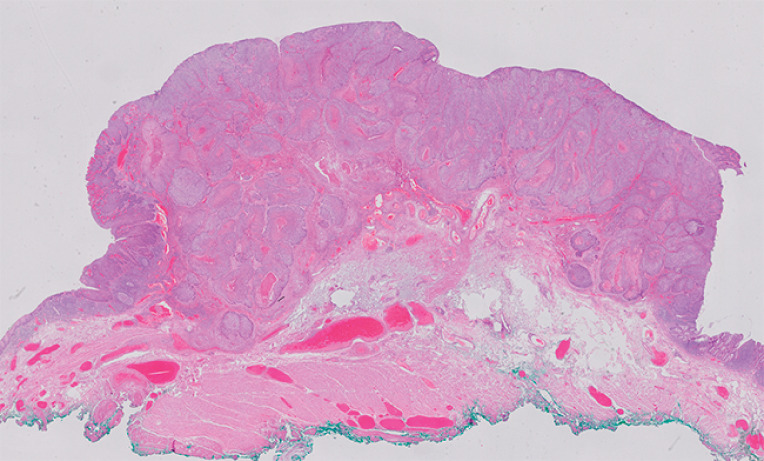
Histological analysis of the resected specimen (hematoxylin-eosin stain) showing a T1Sm2 squamous cell carcinoma with free resection margins (>2 mm).

Endoscopic intermuscular dissection of early anal cancer: step-by-step demonstration of the procedure.Video 1

In conclusion, EID is a feasible and potentially curative treatment option for small, localized early-stage anal cancers.

Endoscopy_UCTN_Code_TTT_1AQ_2AD_3AF

## Notes

Endoscopy E-Videos https://eref.thieme.de/e-videos
*E-Videos* is an open access online section of the journal *Endoscopy* , reporting on interesting cases and new techniques in gastroenterological endoscopy. All papers include a high-quality video and are published with a Creative Commons CC-BY license. Endoscopy E-Videos qualify for HINARI discounts and waivers and eligibility is automatically checked during the submission process. We grant 100% waivers to articles whose corresponding authors are based in Group A countries and 50% waivers to those who are based in Group B countries as classified by Research4Life (see: https://www.research4life.org/access/eligibility/ ). This section has its own submission website at https://mc.manuscriptcentral.com/e-videos .
